# Identification and Mapping of a 2,009-bp DNA Deletion in* SERPING1* of a Hereditary Angioedema Patient

**DOI:** 10.1155/2019/7052062

**Published:** 2019-02-24

**Authors:** Wai-Yu Wong, Helen Wong, Elaine Au, Eric Chan

**Affiliations:** Division of Clinical Immunology, Department of Pathology, Queen Mary Hospital, Hong Kong

## Abstract

We report a heterozygous, 2,009 base pairs (bps) genomic DNA deletion within the* SERPING1* gene that has not previously been reported in a case of type I hereditary angioedema (HAE). The patient is a 28-year-old Han Chinese female living in Hong Kong who has suffered from recurrent angioedema since adolescence, with increasing attack frequency as she entered adulthood; in the past, episodes occurred annually, but now occur every two to three months. The affected areas are not itchy and include common sites such as the left and right forearms, but without throat involvement. The patient also experiences epigastric pain. The patient's mother suffers from similar symptoms. A mutation in the serine protease inhibitor, clade G, member 1 (*SERPING1*) gene is associated with HAE. Patients with HAE type I commonly carry either a small deletion within* SERPING1* or a truncated transcript. We performed a multiplex ligation-dependent probe amplification (MLPA) assay on our indexed patient. Our result suggests a 2,009 bps deletion spanning across exons 5 and 6 within* SERPING1*. Although earlier literature has described other large DNA deletions encasing exons 5 and 6 in* SERPING1*, these DNA rearrangements were larger in size between 4 and 6 kbps, and the breakpoint locations were generally not determined due to technical constraints (Pappalardo et al., 2000; Duponchel et al., 2001; Roche et al., 2005; Loules et al., 2018; and Gö*β*wein et al., 2008). Our report describes mapping of this 2,009 bps in* SERPING1*. Using a combination of molecular techniques, we were able to confirm and locate this large heterozygous genomic DNA deletion that includes both exons 5 and 6 of* SERPING1*.

## 1. Introduction

Hereditary angioedema (HAE) is a rare inborn disorder characterized by edema in different parts of the body, including the limbs, face, and throat [1]. The disease can be potentially life-threatening when swelling obstructs the airway. During attacks, patients may suffer from severe abdominal pain and nausea that are caused by inflammation in the intestinal wall. Episodes can be triggered by various factors, including stress and medications.

HAE can be broadly divided into two groups, with the first group being associated with C1 inhibitor (C1-INH) deficiency (i.e. C1-INH-HAE), while the second group is independent of C1-INH abnormality and is commonly referred to as “HAE with normal C1-INH” [2, 3]. In the former, C1-INH-HAE can be further subcategorized into types I and II and they are clinically indistinguishable, which can only be differentiated by laboratory testing. C1-INH-HAE is an autosomal-dominant disease and occurs with an average frequency of about 1 in 50,000 [3–5]. Type I and type II HAE are due to mutations in the serine protease inhibitor, clade G, member 1 (*SERPING1*) gene, which encodes for the C1-INH protein, a protease inhibitor belonging to the serpin superfamily with its main function to inhibit complement C1 and plasma kallikrein [6–8]. Activation of prekallikrein leads to generation of bradykinin (i.e., a potent vasodilator) that causes angioedema attacks [9]. Both types of HAE are caused by heterozygous mutations in* SERPING1* on chromosome 11q. Though very rare, homozygous mutations in the* SERPING1* gene have also been documented [10–13]. The majority of HAE patients are type I (80-85%) and generally exhibit serum C1-INH levels that are 35% less than normal [14]. In type II HAE, C1-INH levels are normal in the serum or even elevated, but the protein is dysfunctional. HAE patients exhibiting normal C1-INH levels and function have also been documented and are collectively described as “HAE with normal C1-INH” [2]. Several gene targets have been identified for this group, including the genes encoding for factor XII [15], angiopoietin-1 [16], plasminogen [17], or unknown. Mutation in these genes results in increase in vascular permeability that causes HAE episodes and can sometimes explain HAE cases that have normal C1-INH levels or function.

The present report describes a case of type I HAE in a 28-year-old Han Chinese woman living in Hong Kong whose mother suffers from similar symptoms. The patient's diagnosis was established by a C1-INH concentration study and functional assay, followed by genetic confirmation using a multiplex ligation probe amplification (MLPA) assay and long-range polymerase chain reaction (PCR). Genomic sequencing of the amplicons allowed mapping of a large DNA deletion of 2,009 bps within* SERPING1* that has not yet been reported, which accounted for the patient's (and her mother's) type I HAE.

## 2. Case Presentation

Our indexed patient is a 28-year-old Han Chinese female living in Hong Kong who has suffered from recurrent episodes of angioedema since adolescence, with an increasing number of attacks as she entered adulthood. These episodes occurred annually in the past, but have now increased to every two to three months. The edemas are not itchy and the affected areas include common swelling sites such as the left and right forearms; there is no throat involvement. The patient also complains about epigastric pain. The patient's mother suffers from similar symptoms (although with greater severity than the patient), suggesting a hereditary component of the patient's disease. The patient's serum C1-INH level (patient: <0.03 mg/mL, reference: 0.224–0.387 mg/mL) and C1-INH function (patient: 0.12 U/mL, reference: 0.7–1.3 U/mL) were both low; attenuation of C1-INH function was expected due to the patient's low serum C1-INH concentration. The patient's C3 level was normal but the C4 level was also low, which could be explained by the loss of C1-INH, which accelerated the consumption of C4. These results collectively indicated a C1-INH deficiency, which manifests in type I HAE.

**Figure 1:**
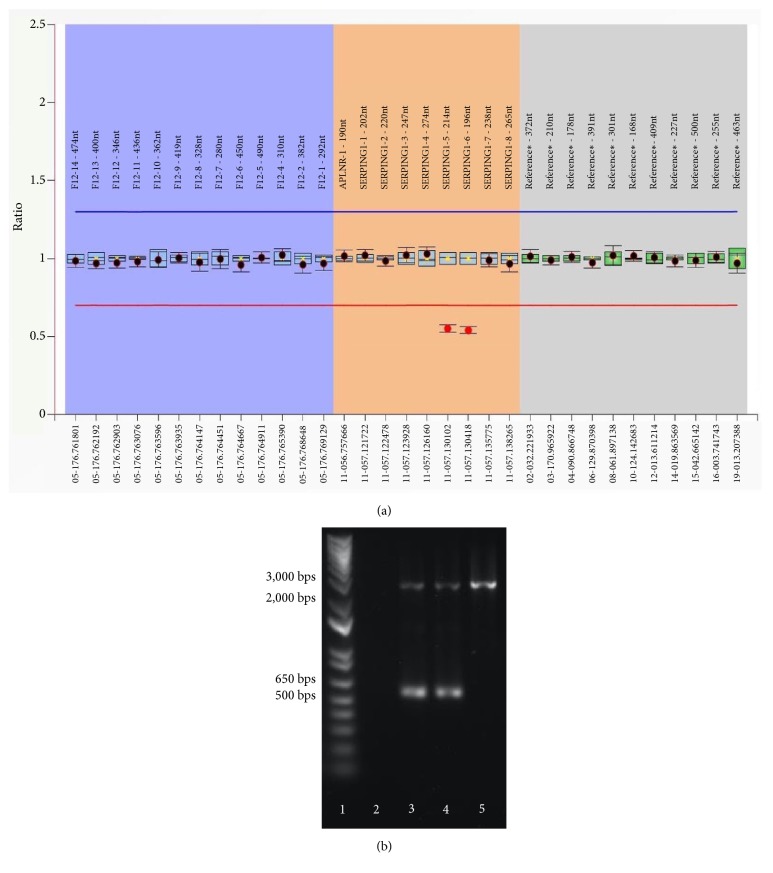
Heterozygous deletion of exons 5 and 6 in the patient's genomic DNA was detected by the MLPA assay (a). The mother had an identical MLPA result (data not shown). The patient and her mother's genomic DNA produced heterozygous PCR products, suggesting an approximately 2,000-bp deletion (b) (Lane 1: DNA ladder; Lane 2: negative control; Lane 3: patient; Lane 4: patient's mother; and Lane 5: wild-type control).

We began analyzing the patient's* SERPING1* gene by Sanger sequencing but found no abnormality; we suspected that our result could be due to a large DNA deletion that may not be detectable by Sanger sequencing since the variant allele would not be amplifiable. To investigate this, we employed the MLPA assay, a sensitive assay that allows the detection of DNA copy number changes of up to 45 loci in one relatively simple, semiquantitative PCR-based reaction. Using this technique, we found that the DNA copy numbers of exons 5 and 6 were half of the other exons in the same* SERPING1* gene (Figure 1(a)), suggesting heterozygous deletions for each of these two exons. Because HAE is an autosomal dominant disorder, our finding of heterozygous* SERPING1* deletion by the MLPA assay corroborated the patient's clinical history.

**Figure 2:**
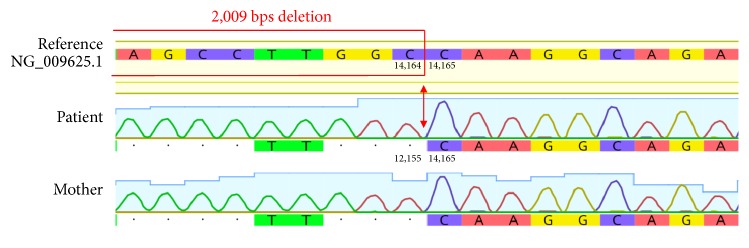
Genomic DNA sequence electropherograms of the patient and her mother, in comparison with the reference sequence. The deletion is 2,009 bps in size and encompasses exons 5 and 6. The red arrows locate the 3′ end breakpoint, whereas the red frame illustrates a part of the deleted sequence. The deletion boundaries are noted on the patient strand, as indicated by the nucleotide positions.

**Figure 3:**
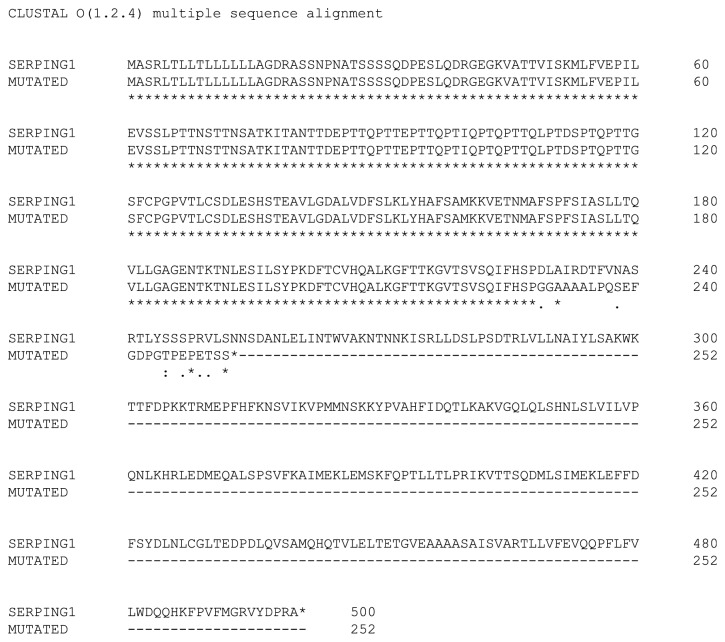
NG_009625.1:g.12156_14164del2009 led to the truncation of a 500-amino acid C1-INH protein into a 252-amino acid dysfunctional protein. Please note the above amino acid sequences relate only to the codons and not the mature protein with post-translational modifications.

The sequences of exons 5 and 6 are both short (204 and 140 bps, respectively). Given their small size and close proximity (they are only 194 bps apart), we deduced that the deletion was most likely a large genomic DNA deletion that spanned across both of these exons (i.e.,* cis* phase), instead of two separate deletions of exons 5 and 6 on different DNA strands (i.e.,* trans* phase). The total length, including the introns before exon 5 and after exon 6, was 9,547 bps. This segment was too large to be amplified by conventional PCR, and, therefore, to confirm the deletion, we used long-range PCR to amplify the segment between exons 4 and 7. As resolved by gel electrophoresis, we observed two PCR products at different lengths; one was at the expected molecular size of approximately 10,000 bps, whereas the other smaller PCR product was approximately 8,000 bps (data not shown). This smaller PCR product was likely contributed by the variant allele with the deletion. Notably, the presence of these two PCR products supported our prediction that the patient carries a large DNA mutation that covers exons 5 and 6 in the* cis *phase, instead of a deletion of exon 5 and a deletion of exon 6 on separate DNA strands, as this would have produced two smaller PCR products instead of one. Unfortunately, Sanger sequencing can only process sequences of approximately 1,000 bps or shorter, so the approximately 8,000-bp PCR product was too large to be directly tested by this approach. In order to precisely locate the boundaries of the deletion, we first designed several primer pairs amongst different regions between exons 4 and 7 to scan for the deletion. One pair of these primers (Supplementary Table 1) produced heterozygous PCR products from the patient's genomic DNA (Figure 1(b)). Using the gel purification method, the smaller PCR product was then isolated and subjected to Sanger sequencing (Figure 2). From this smaller PCR product, which was anticipated to be from the variant allele, we were able to determine that the deletion was 2,009 bps long and between positions 12,156 and 14,164 on the genomic DNA (i.e., NG_009625.1:g.12156_14164del2009). This large genomic DNA deletion has lost both exons 5 and 6, leading to the truncation of a 500-amino acid protein into a 252-amino acid protein (i.e., a deletion of 272 amino acids and substitution of 24 nonsense amino acids) (Figure 3). This variation is considered to be “pathogenic” according to the ACMG 2015 guidelines [18]. Although some reports have discussed deletions of exon 5 and/or exon 6, these reported deletions were larger, approximately 4-6 kbps in size [19–23], and this particular 2,009 bps deletion variant encompassing exons 5 and 6 that we have detected has not yet been previously described. Essentially, our molecular findings explained the cause of the patient's low C1-INH level and function.

The patient's mother suffers from similar symptoms (but with greater severity in comparison to the patient) and displays laboratory findings that are comparable to those of the patient (i.e., serum C1-INH level, mother: <0.03 mg/mL, reference: 0.224–0.387 mg/mL; and C1-INH function, mother: 0.09 U/mL, reference: 0.7–1.3 U/mL). The mother's genomic DNA was also subjected to the MLPA assay and Sanger sequencing, and the same mutation found in the patient was also detected in the mother (i.e., NG_009625.1:g.12156_14164del2009), indicating that the patient's mutation was inherited from the mother and that the detected mutation is* not de novo* for the patient. However, whether the mother's mutation is* de novo* remains inconclusive as samples from the grandparents or the mother's siblings were not available for further investigation.

## 3. Discussion

*SERPING1* mutations are highly heterogeneous and until the writing of this report, more than 500 mutations have been described for this gene (http://www.hae.enzim.hu, https://www.ncbi.nlm.nih.gov/clinvar/ and http://www.hgmd.cf.ac.uk/ac). The majority of the reported cases (i.e., approximately 70-75%) had point mutations that led to missense mutations or short insertions/deletions that caused frame-shift alterations, whereas larger structural mutations, such as indels, make up around 15-20% of all cases. Finally, the remainder 10% was splice-site and regulatory mutations. The data presented in this study illustrate the identification of a large DNA deletion of 2,009 bps that is carried by both the patient and her mother and provide insight into the etiology of their type I HAE.

**Figure 4:**
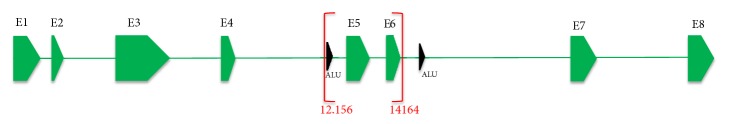
NG_009625.1:g.12156_14164del2009 in* SERPING1* (red brackets), and the nearest Alu element sequences (black arrows) relative to this 2,009 bps deletion. The 5′ end of the deletion breakpoint is 16 bps from the adjacent Alu element, while the 3′ end is approximately 1 kbps away from the nearest Alu sequence. Please note the diagram is not in exact scale.

The gene* SERPING1* contains high density of repeating unit called “Alu element”, a transposable sequence that is known to facilitate deletions through insertion-mediated deletion or recombination-associated deletion [24, 25]. Because most of the Alu elements are found in introns 4 and 6 [14], we also studied whether exons 5-6 deletion that we have detected could be related to these Alu elements. We located the surrounding Alu sequences around NG_009625.1:g.12156_14164del2009, but neither 5′ nor 3′ ends of the deletion breakpoints are intersected by Alu element sequences. The 5′ end of the deletion breakpoint is 16 bps from the nearest Alu element, but the 3′ end is approximately 1 kbps away from the adjacent Alu sequence (Figure 4). Given the remoteness of the Alu sequence on the 3′ end of the deletion breakpoint, whether Alu elements are involved in the formation of the NG_009625.1:g.12156_14164del2009 deletion remains elusive, and the mechanism causing this 2,009 bps deletion will require further investigation.

The truncation of exons 5 and 6 (which together encode a total of 115 amino acids) evidently has devastating consequences for the expression of C1-INH and subsequently its function. The deleterious effects of these large heterozygous deletions involving exon 5 and/or exon 6 are supported by other similar mutations that were previously found in HAE patients [19–23]. Interestingly, most of these earlier reports that described large deletions affecting exon 5 and/or exon 6 did not pinpoint the breakpoint positions of these deletions, but instead only the approximate sizes of the deletions were noted and these deletions also appeared larger in size than our detected variant (i.e., 4-6 kbps) [19–23]. The extensions of these deletions that were not further evaluated in these cohorts were due to technical and resource constraints. In fact, dissecting large DNA mutation is often challenging, as the process to assess even just one case can be time-consuming, labor intensive, and expensive. Obviously, technology has evolved and having access to newer methods, such as next-generation sequencing (NGS), should facilitate the investigation of the boundary positions of such mutations. A recent report from Loules et al. discussed the implementation of a custom NGS platform for sequencing the whole* SERPING1* gene in a clinical setting [22]. However, in addition to remaining relatively expensive, NGS also requires highly specialized personnel for operation and data analysis, which makes it difficult to implement in most clinical laboratories. Moreover, NGS is not perfect; it has also been reported to have difficulty in detecting intermediate-sized deletions and insertions [26]. Alternatively, an approach named exon quantification technique (EQT) for the detection of large genetic rearrangements in* SERPING1* has also been described [27]. This method is based on the Quantitative Multiplex PCR Short Fluorescent Fragment method that was designed to enhance the search of large indels that could be difficult to be detected by Sanger sequencing [20]. The authors discussed the EQT assay to be a sensitive, cheap, quick, and more direct method for study of large DNA alterations and appear as an attractive approach for investigation of large indels in* SERPING1*. In our case, we have spent several months testing many pairs of primers by conventional PCR and Sanger sequencing to dissect the deletion. Importantly, our report is the first to have precisely defined the boundaries of a 2,009 bps deletion surrounding exons 5 and 6 of* SERPING1*.

For our indexed patient and her mother, although both of them share the same genetic mutation, their disease progressions appear to differ, with the mother showing more severe symptoms including bowel edema and episodes of throat swelling, where these symptoms are absent in the indexed patient. Perhaps it has to do with their age difference, but the exact reason as to why the patient and her mother are not showing the same degree of disease phenotype remains unclear. It has been documented in earlier literature that clinical expression of HAE for the same* SERPING1* mutation can vary considerably, suggesting that other factors besides mutation of* SERPING1* contribute to the diversity of clinical manifestations [8]. Moreover, several studies have attempted to determine correlation between clinical phenotypes and* SERPING1* gene mutations; however, the results were largely conflicting [1, 8, 16, 28, 29], and the attempt to explain clinical presentation based on* SERPING1* mutation has not been successful. Other factors such as environmental stimuli, hormonal effects, and epigenetic changes have been suggested to interact with the disease genotype to direct disease manifestation. Furthermore, the level of kinin catabolism has also been implicated as a potential predictive parameter of HAE severity [30]. Hence, the exact mechanism in causing HAE symptoms remains obscure and will require further examination.

Discovery of this large DNA deletion required a mixture of different molecular approaches. Another learning point from our study is that this gross DNA mutation would not have been detectable with only Sanger sequencing, a widely used method that is often considered the gold standard in genetic studies. Sanger sequencing is ineffective in delineating long stretches of repetitive sequences, is unreliable in detecting large DNA mutations, and can only work with small DNA fragments of approximately 1,000 bps. In our case, successful genetic examination required the correct use of other molecular techniques, such as the MLPA assay, and appropriate primer combinations to properly define the 2,009-bp DNA deletion. Investigators need to be fully aware of the strengths and limitations of their methodologies to accurately detect certain kinds of genetic abnormalities.

In conclusion, our results have further expanded the spectrum of known* SERPING1* mutations and will contribute to a better understanding of C1-INH-HAE.

## Supplementary Materials

Supplementary Materials This section describes the methods and materials used in this study, including the primer sequences in the various PCRs.
