# Supplementary material for: Identification and Mapping of a 2,009-bp DNA Deletion in SERPING1 of a Hereditary Angioedema Patient
Source: Case Rep Genet. 2019 Feb 24;2019:7052062. doi: 10.1155/2019/7052062 (PMC6409050; doi:10.1155/2019/7052062)
Supplement: Supplementary Materials — This section describes the methods and materials used in this study, including the primer sequences in the various PCRs. [file 7052062.f1.pdf]

Supplementary Information

Methods and materials

DNA extraction

Genomic DNA was extracted from blood samples by a QIAamp® DNA Blood Mini Kit (Qiagen). DNA purity was assessed using a Nanodrop™ spectrophotometer (Thermo Fisher Scientific); only samples with 260:280 ratios between 1.8 and 2.0 were used in order to ensure high sample quality for the subsequent procedures.

Sanger sequencing and data analysis

PCR fragments were sequenced on an ABI 3100 (Applied Biosystems) automatic sequencer. These PCR fragments were purified using an ExoSAP-IT (USB) and sequenced in the forward and reverse directions using Big-Dye Terminator chemistry (Applied Biosystems). Sequencing data were analyzed using the Geneious® 11.0.5 software.

Multiplex ligation probe amplification (MLPA) Assay

We used the SALSA MLPA probemix P243-B1 SERPING1-F12 (MRC-Holland MLPA®) for the MLPA assay and the ligation and amplification steps were performed in a C1000™ thermal cycler with a heated lid (Bio-Rad). The P243-B1 SERPING1-F12 probemix contains 33 MLPA probes that target all the exons of SERPING1. The amplified probes were detected on an ABI 3100 (Applied Biosystems) automatic sequencer. Analyses were performed with the Coffalyser.Net software (MRC-Holland). Each individual probe-relative area was compared with the corresponding

probe-relative areas of 3 control samples, thus creating a ratio (case/control) for the final results.

Long-range PCR

A segment of SERPING1 enclosing exons 4-7 was amplified using the forward primer SERPING1-Ex4f (Supplementary Table 1) and the reverse primer SERPING1-Ex7r. The total reaction volume was 50 µL, which included the Expand Long Template PCR System (Roche Applied Science) with 350 µM of dATP, dGTP, dCTP, and TTP, 0.5 µM of each primer, 0.75 µL of DNA polymerase mix, and 500 ng of DNA. After 2 min at 94°C, the cycling conditions were: 10 cycles at 94°C for 10 sec, 65°C for 30 sec, and 68°C for 8 min. This was followed by 25 cycles at 94°C for 15 sec of denaturation, 65°C for 30 sec of annealing, and 68°C for 8 min of elongation but with an addition of 20 extra seconds for each successive elongation cycle. The final extension was at 68°C for 7 min.

PCR-amplification

PCR using the forward primer SERPING1-X1FA and reverse primer SERPING1-2550R (Supplementary Table 1) was performed in a 25-µL total reaction volume using the GoTaq® G2 Hot Start Polymerase kit (Promega), which contained 5 µL of ColorLess GoTaq® Flexi Buffer (Promega), 2.5 µL of 25 mM Magnesium Chloride (Promega), 1 µL of 10 mM PCR nucleotide mix (Roche), 0.5 µL of each primer (10 µM), 10 µL of DNA extract (final conc. 100 ng), 0.25 µL (5 units/µL) of GoTaq® G2 Hot Start Polymerase (Promega), and 5.25 µL of deionized water. The thermal

cycling conditions were: 95°C hold for 5 min; 30 cycles of 95°C for 30 sec, 60°C for 30 seconds, and 72°C for 90 sec; and a 72°C hold for 7 minutes. A negative water control and a wild-type control were included for quality control purposes.

Agarose gel electrophoresis of DNA

One µL of 6X DNA Gel Loading Dye (Thermo Fisher Scientific) was mixed with 5 µL of the DNA sample and electrophoresed at 80-100 V on a 0.8 – 1.0% agarose gel in TAE. After electrophoresis, the agarose gel was stained in 1X Gel-Red (Biotium) in TAE for 30 min. The DNA bands were visualized using the Gel Documentation System (Major Science).

Gel purification of DNA

DNA was purified from TAE agarose gels using the QIAquick® Gel Extraction Kit (QIAGEN). Briefly, after cutting the DNA band out of the gel, the gel that contained the band was dissolved at 50°C in buffer solution with isopropanol. The dissolved mixture was passed through a mini-column from the kit and the liquid that passed through was discarded. The column was washed by the provided wash solution. Finally, elution buffer (50 µL) was added directly to the mini-column to elute the DNA.

Supplementary Table 1: Primers used in this study.

Primer Name	Direction	Sequence (5’ -> 3’)	Targeted Location
SERPING1-Ex1f*	Forward	GGCCAGCCAATAGCTAAGAC	Exon 1
SERPING1-Ex1r*	Reverse	ACCCCCTCCCTAGACCTCTT	Exon 1
SERPING1-Ex2f*	Forward	GGAGGGAATTCGCTAAGAGG	Exon 2
SERPING1-Ex2r*	Reverse	CGGAGCCTGAAGGGTTAAT	Exon 2
SERPING1-Ex3f*	Forward	GGACTGTGCCTCGTAGTAAGAAA	Exon 3
SERPING1-Ex3r*	Reverse	TGGGAGTGTCCAACAAATGA	Exon 3
SERPING1-Ex4f*#	Forward	AACCCTCATTCCCAAGGAAG	Exon 4
SERPING1-Ex4r*	Reverse	TTCCCTCTGTCCTTTCTTTCC	Exon 4
SERPING1-Ex5f*	Forward	TCAAATCGTGCTCATGGAAA	Exon 5
SERPING1-Ex5r*	Reverse	CTTGGGGTTAAGTGGGCTTT	Exon 5
SERPING1-Ex6f*	Forward	GGATCTCAATGTCCCTGCAC	Exon 6
SERPING1-Ex6r*	Reverse	CCCCAAAATGATGGGACTAC	Exon 6
SERPING1-Ex7f*	Forward	CAGGAGAGAGATGCGGTAGG	Exon 7
SERPING1-Ex7r*#	Reverse	CCTCTAACTTGCAGGGTTGC	Exon 7
SERPING1-Ex8f*	Forward	GGACAAAGGTCTCCATCAGC	Exon 8
SERPING1-Ex8r*	Reverse	AACTGAGAGCTGAGGCTGGA	Exon 8
SERPING1-X1FA	Forward	CCTCAGCCAGGGTCCATTTT	NG_009625.1:g.11983_14533<br>(i.e. regions surrounding Exons 5-6)
SERPING1-2550R	Reverse	TTGCCAGTTGGGATCCTCTG	NG_009625.1:g.11983_14533<br>(i.e. regions surrounding Exons 5-6)

*Referenced from El-Meguid AM, Aslanidis C, Schimanski S, Schambeck C, Schmitz G. New mutations in C1 esterase inhibitor (SERPING1) in a German family with hereditary angioedema. The Egyptian journal of immunology. 2008;15: 93.

#These primers were used in the long-range PCR
